# Phytoestrogens and Their Metabolites in Bulk-Tank Milk: Effects of Farm Management and Season

**DOI:** 10.1371/journal.pone.0127187

**Published:** 2015-05-21

**Authors:** Steffen A. Adler, Stig Purup, Jens Hansen-Møller, Erling Thuen, Håvard Steinshamn

**Affiliations:** 1 Bioforsk—Norwegian Institute for Agricultural and Environmental Research, 6630, Tingvoll, Norway; 2 Department of Animal and Aquacultural Sciences, Norwegian University of Life Sciences, 1432, Ås, Norway; 3 Department of Animal Science, Aarhus University, Foulum, 8830, Tjele, Denmark; Graz University of Technology (TU Graz), AUSTRIA

## Abstract

Phytoestrogens have structures similar to endogenous steroids and may induce or inhibit the response of hormone receptors. The objectives of the present study were to compare the effects of long-term vs. short-term grassland management in organic and conventional dairy production systems, compare organic and conventional production systems and assess seasonal variation on phytoestrogen concentrations in bulk-tank milk. The concentrations of phytoestrogens were analyzed in bulk-tank milk sampled three times in two subsequent years from 28 dairy farms: Fourteen organic (ORG) dairy farms with either short-term or long-term grassland management were paired with 14 conventional (CON) farms with respect to grassland management. Grassland management varied in terms of time since establishment. Short-term grassland management (SG) was defined as establishment or reseeding every fourth year or more often, and long-term grassland management (LG) was defined as less frequent establishment or reseeding. The proportion of red clover (*Trifolium pretense* L.) in the herbage was positively correlated with milk concentrations of the mammalian isoflavone equol. Therefore, organically produced bulk-tank milk contained more equol than conventionally produced milk, and milk from ORG-SG farms had more equol than milk from ORG-LG farms. Milk produced during the indoor-feeding periods had more equol than milk produced during the outdoor feeding period, because pastures contained less red clover than fields intended for silage production. Organically produced milk had also higher concentrations of the mammalian lignan enterolactone, but in contrast to equol, concentrations increased in the outdoor-feeding periods compared to the indoor-feeding periods. There were no indications of fertility problems on ORG-SG farms who had the highest red clover proportions in the herbage. This study shows that production system, grassland management, and season affect milk concentrations of phytoestrogens. However, compared to soy products, milk concentrations of phytoestrogens are low and future studies are required to investigate if the intake of phytoestrogens from dairy products has physiological effects in humans.

## Introduction

Phytoestrogens are phenolic, plant derived compounds and divided into several groups, including isoflavones, lignans and coumestans. In plants, phtyoestrogens have various functions such as defense against pathogens [1]. Phytoestrogens have structures similar to endogenous steroids and may induce or inhibit the response on hormone receptors in animals or humans. In sheep, high intake of phytoestrogens, especially formononetin has been found to impair fertility [2, 3], whereas in cattle, effects are not consistent [3–6]. In humans, dietary phytoestrogens may protect against cancers or prevent osteoporosis and some may function as antioxidants. The most important source of phytoestrogens in the human diet are soy products [7], however, the intake of animal derived phytoestrogens may be significant [8].

The isoflavones, formononetin and daidzein, are to a large extent converted by rumen microorganisms to the mammalian isoflavone equol [9]. Equol has a much higher estrogen activity than daidzein [10–12]. Biochanin A and genistein can be converted to substances with no estrogenic activity [7, 13]. The lignans secoisolariciresinol and matairesinol can be converted by intestinal microorganisms to enterodiol and enterolactone [14]. However, a recent study has shown that also ruminal *Prevotella* spp. can metabolize secoisolariciresinol to enterolactone [15]. Also other lignans such as pinoresinol, syringaresinol and lariciresinol are precursors of enterodiol and enterolactone [16].

Grassland management and production system affect sward botanical composition [17]. Therefore, it is likely that botanical composition affects the uptake of phytoestrogens and concentrations in milk. Red clover (*Trifolium pratense* L.) has high concentrations of formononetin [18], and several studies have shown high concentrations of equol in milk from cows fed silage containing red clover [19–21], or from cows grazing pastures containing red clover [22, 23]. Elevated levels of equol have also been reported from organically produced consumer milk compared to conventional milk [24, 25].

Less is known about the precursors of the mammalian lignans enterodiol and enterolactone. In the study by Höjer et al. [21], feeding silage produced from short-term grassland resulted in milk that contained less enterolactone than silage from long-term grassland, whereas in the study by Adler et al. [22] grazing a short-term grassland gave higher enterolactone concentration in milk than grazing a long-term pasture.

Although the above-cited studies indicate specific effects of botanical composition on phytoestrogen concentrations in milk, to our knowledge no attempts have been made so far to investigate the effect of botanical composition at the level of farming systems.

The objectives of the present study were to compare the effects of long-term vs. short-term grassland management in organic and conventional production systems, compare organic and conventional production systems and assess seasonal variation on phytoestrogen concentrations in bulk-tank milk.

## Material and Methods

### Experimental Design

Twenty-eight dairy farms in central-Norway participated in the study in 2007 and 2008 and have been previously described by Adler et al. [17]. In brief, 7 organic (ORG) farms with short-term grassland management (SG), referred to as ORG-SG-farms, were paired with 7 conventional (CON) farms with SG, referred to as CON-SG-farms, and 7 ORG-farms with long-term grassland management (LG), referred to as ORG-LG-farms, were paired with 7 CON-farms with LG, referred to as CON-LG-farms. Grassland management was defined as SG when the grassland fields of a farm were renewed every fourth year or more frequently and as LG when the fields were renewed less frequently. Grassland fields were renewed by soil tillage and seeding. Organic and conventional farms were paired on location and calving pattern, based on information from local extension services. The organic farms were certified by the Norwegian certification body Debio (Bjørkelangen, Norway) according to the EU standards for organic farming [26]. In brief, the standards for organic farming require a minimum forage intake in total dry matter intake (DMI; 50% in the first 3 months of lactation increasing to 60% thereafter) and all feeds have to be grown organically, i.e. without use of synthetic pesticides and synthetic N-fertilizers. Fertilization with animal manure is limited to 170 kg N/ha and yr. All farms participated in the Norwegian Dairy Herd Recording System and delivered milk to the same dairy company (TINE Norwegian Dairies SA, Oslo). On all farms, forages were fed ad libitum and allocated concentrate amounts were based on individual milk yields.

### On Farm Analysis, Sampling and Data Collection

Data on farm characteristics were collected in farmer interviews and milk production data were collected from the Norwegian Dairy Herd Recording System. On average, the dairy farms had a farmland area of 28 ha (SD 9.6), a herd size of 19.6 dairy cows (7.9 SD), a forage area proportion of 0.86 (SD 0.150) for SG and 1.00 (SD 0.017) for LG, and a grassland age of 3 (SD 0.9) yr for SG and 11 (SD 3.8) yr for LG [17].

Herbage botanical composition before first cut silage in 2007 was estimated on 4 selected fields on each farm by the dry-weight-rank method [27], modified by Jones and Hargreaves [28]. The selected fields represented overall grassland use including fields that were cut, cut and grazed in combination, or only grazed.

In February, June and October in 2007, and February, June, August and October 2008, milk was sampled from stirred bulk-tanks and the samples were transported chilled (4°C) to the dairy.

### Animals and Diets

On most farms cows were in tie-stalls and on 6 farms, mainly SG-farms, in loose-house barns. The cows were mainly fed grass silage based diets during the indoor-feeding periods (October to mid-May), and on all farms cows grazed in the outdoor-feeding periods, although many herds also had access to silage. Only herds with Norwegian Red dairy cows were included in the study, and calving time was rather evenly distributed over the year on all farms. Dairy feed rations included forage, concentrates, mineral mixtures and vitamin mixtures on all farms. Silage fermented in bulk silos or in round bales was the main forage in the indoor-feeding periods. On most farms the herbage was wilted before ensiling. In brief, the silages fed on ORG farms had had lower concentrations of crude protein (ORG: 138 g/kg of dry matter (DM), CON: 169 g/kg of DM), but higher concentrations of non-fibrous carbohydrates (ORG: 219 g/kg of DM; CON: 175 g/kg of DM). Neutral detergent fiber concentration (mean: 570 g/kg of DM), in vitro digestibility (mean: 813 g/kg of DM) and net energy of lactation (NEL) (mean: 5.7 MJ/kg of DM) did not differ [17]. Pasture samples and concentrate samples were not analyzed for chemical composition. The cows were kept indoors at night on 8 farms (ORG-SG: 1, ORG-LG: 3, CON-SG: 1, CON-LG: 3) during the outdoor-feeding periods. Homegrown grains of barley and oats were fed in addition to commercial concentrates on most ORG-SG farms and supplemented with fishmeal on 2 ORG-SG farms.

Commercial concentrates were used on all farms, but ingredients varied; organic mixtures (on average) contained: barley 29%, wheat 25%, oats 21%, fishmeal 7%, sugarcane molasses 5%, expeller soybean meal 4%, and conventional mixtures (on average) contained: barley 36%, oats 15%, solvent-extracted soybean meal 12%, Sorghum (*Sorghum bicolor* (L.) Moench) 10%, rape seeds and expeller rape seed meal 8%, sugar beet pulp 8%, sugarcane molasses 7%, rumen protected fat (AkoFeed Gigant 60; Aarhus Karlshamn AB, Malmö, Sweden) 2%, vegetable fat (AkoFeed Standard; Aarhus Karlshamn AB) 1%). On some farms also other feed supplements such as potato (1 ORG-SG farm), whey (1 ORG-LG farm, 1 CON-SG farm), brewers’ grain (1 CON-SG farm) or macro algae meal were used (1 ORG-SG farm).

### Analysis Methods

#### Sample Preparation and Chemical Analysis of Milk

Milk samples intended for analysis of gross composition, urea, and FFA were preserved with 2-bromo-2-nitropropane-1,3-diol (Bronopol, D&F Inc., Dublin, CA) and analyzed by a Fourier transformed infrared spectroscopy milk analyzer (MilkoScan 6000 FTIR, Foss, Hillerød, Denmark). Milk samples intended for analysis of phytoestrogens were frozen at -20°C immediately after arriving at the dairy.

Phytoestrogen concentrations in herbage, concentrate and milk were analyzed according to the methods described by Steinshamn et al. [19]. Briefly, herbage samples were extracted with ethanol and acetate buffer (pH 5.0), then incubated with Cellulase Onozuka R-10 from *Trichoderma viride* (Merck, Darmstadt, Germany) at room temperature overnight, followed by centrifugation. Milk samples were defatted and deproteinized by mixing with acetate buffer (pH 5.2), heptane and acetone. The acetone/water phase was separated and evaporated to dryness and residues re-dissolved in water. Conjugates of phytoestrogens were cleaved by incubation with β-glucuronidase and sulfates from Helix pomatia type H2 (Sigma/Aldrich, St. Louis, MO) at 40°C for 4 h followed by centrifugation. Secoisolariciresinol, matairesinol, enterodiol, daidzein, enterolactone, equol, genistein, coumestrol, formononetin, prunetin and biochanin A were analyzed using the liquid chromatography (LC)-mass spectrometry (MS)/MS technique (Micromass, Manchester, UK) with standard addition [19].

### Calculations and Statistical Analysis

Data of the participating herds were collected from the Norwegian Dairy Herd Recording System. Milk gross composition is the weighted average of all individual milk samples analyzed in one year on each farm. Energy corrected milk (ECM) yields were calculated as kilograms of milk per cow and year × (0.01 + 0.0122 × g of fat/kg of milk + 0.0077 × g of protein/kg of milk + 0.0053 × g of lactose/kg of milk).

Daily concentrate intake for individual cows was forwarded by the farmers to the Norwegian Dairy Herd Recording System and mean concentrate intake per year and cow was calculated. Forage intake of NEL per cow and year was estimated by the Norwegian Dairy Herd Recording System as net energy requirement for maintenance, production, activity and pregnancy and subtracting NEL supplied from concentrates. The cow fertility in the participating herds was evaluated by three indicators, age at first calving, calving interval and animals culled due to fertility problems per cow and year. All data were based on the Norwegian Dairy Herd Recording System.

If the content of phytoestrogens was below the limit of detection, half the detection limit was used to be able to run the statistical analysis. The limits of detection in milk were 0.391 μg/kg for secoisolariciresinol, 0.021 μg/kg for matairesinol, 0.018 μg/kg for enterodiol, 0.072 μg/kg for daidzein, 0.067 μg/kg for enterolactone, 0.040 μg/kg for equol, 0.092 μg/kg for genistein, 0.053 μg/kg for coumestrol, 0.012 μg/kg for formononetin, 0.014 μg/kg for prunetin and 0.023 μg/kg for biochanin A.

Milk concentrations of phytoestrogens were analyzed using the MIXED model procedure by SAS [29]. The statistical model (1) was used:

$${\text{Y}}_{\text{ijklmn}}=\mu +{\text{G}}_{\text{i}}+\text{P}{\left(\text{G}\right)}_{\text{ij}}+{\text{M}}_{\text{k}}+{\left(\text{GM}\right)}_{\text{ik}}+{\left(\text{MP}\left(\text{G}\right)\right)}_{\text{ijk}}+\text{f}{\left(\text{G},\text{P}\right)}_{\text{ijl}}+\text{t}{\left(\text{G}\right)}_{\text{im}}+{\text{e}}_{\text{ijklmn}},$$ (1)

where Y were the individual dependent variables for individual phytoestrogen concentrations in milk (n = 1 to 168) and μ was the average of all observations, G was the fixed effect of grassland management (i = 1, 2; where 1 = SG and 2 = LG), P(G) was the fixed effect of production system within G (j = 1, 2; where 1 = ORG and 2 = CON), M was the fixed effect of month (k = 1 through 6; where 1 = February 2007, 2 = June 2007, 3 = October 2007, 4 = February 2008, 5 = June 2008, 6 = October 2008), (GM) and (MP(G)) were interactions of the fixed effects, f was the random effect of farm within G and P (l = 1 through 28), and t was the random effect of farm pair within G (m = 1 through 14) and e_ijklmn_ were the random residual errors, assumed to be independent and N(0, σe2). Observations for month within farm were treated as repeated observations. Contrasts were calculated for the effects of ORG-SG vs. ORG-LG, CON-SG vs. CON-LG, ORG vs. CON and indoor (k = 1, 3, 4, 6 vs. outdoor-feeding periods (k = 2, 5). Differences between means were tested with the Tukey-Kramer test.

In order to find correlations between proportions of botanical families in the herbage, concentrate DMI and milk concentrations of phytoestrogens a principal component analysis was performed by the PRINCOMP procedure in SAS [29]. Average milk concentrations of phytoestrogens and concentrate DMI were calculated for indoor-feeding and outdoor-feeding periods. The botanical composition of fields that were cut or both cut and grazed were associated with the indoor-feeding periods, whereas the botanical composition of fields that were grazed or both cut and grazed were associated with the outdoor-feeding periods.

## Results

### Botanical Composition of Diets

Fields on CON-farms that were cut or cut and grazed in combination had higher proportions of grasses in the herbage (mean 905 g of DM/kg of DM) than fields that were grazed only (mean 670 g of DM/kg of DM) (Table 1). Compared with CON-farms, ORG-farms had lower proportions of grasses (mean 574 g vs. 826 g of DM/kg of DM) and differences between fields that were cut, cut and grazed in combination, or only grazed were small. The prevailing grass species were timothy (*Phleum pratense* L.) and meadow fescue (*Festuca pratensis* Huds.) in all management systems. Smooth meadowgrass (*Poa pratensis* L.) was most common on fields on ORG-SG and CON-SG farms that were grazed only. Red clover was mainly found on fields on ORG-SG and ORG-LG farms that were cut or cut and grazed in combination. In fields that were grazed only, white clover (*Trifolium repens* L.) was the prevailing legume species. The prevailing species belonging to other plant families were northern dock (*Rumex longifolius* DC.) and dandelion (*Taraxacum* spp.). Other botanical families than grasses and legumes were found in higher proportions in fields that were grazed only than other fields. Fields on ORG-LG farms had the highest proportions of these plant families.

**Table 1:** Botanical composition estimated before first cut in 2007 on fields, which were cut, cut and grazed in combination or only grazed on dairy farms with organic production system (ORG) and short-term (ORG-SG) or long-term grassland management (ORG-LG) and dairy farms with conventional production system (CON) and short-term (CON-SG) or long-term grassland management (CON-LG).

Species, g of DM/kg of DM or item	ORG-SG			ORG-LG			CON-SG			CON-LG			SEM		
	Cut	Cut/grazed	Grazed	Cut	Cut/grazed	Grazed	Cut	Cut/grazed	Grazed	Cut	Cut/grazed	Grazed	Cut	Cut/grazed	Grazed
n	16	5	7	19	4	6	18	4	6	20	2	7			
Grasses (*Poaceae*)	628	656	632	491	521	515	917	961	675	817	924	664	57.6	49.0	67.1
Timothy (*Phleum pratense* L.)	337	349	110	124	127	18	501	438	77	248	270	106	51.3	142.6	58.5
Meadow fescue (*Festuca pratensis* Huds.)	73	187	43	80	126	70	142	156	124	141	136	41	43.7	53.5	33.8
Perennial ryegrass (*Lolium perenne* L.)	80	34	49	35	17	62	123	35	77	27	10	44	38.7	17.6	34.5
Smooth meadowgrass (*Poa pratensis* L.)	58	115	148	40	56	97	14	171	263	50	5	89	28.7	130.1	64.1
Rough meadowgrass (*Poa trivialis* L.)	19	79	53	41	58	30	25	40	46	118	262	143	27.2	49.9	36.7
Common couch (*Elytrigia repens* L.)	36	23	9	8	6	0	65	53	25	112	91	66	29.8	30.0	18.1
Other grasses	25	8	220	168	131	249	39	28	59	121	151	206	40.1	41.3	78.7
Legumes (*Fabaceae*)	270	244	60	144	134	76	35	16	68	16	0	8	25.3	76.5	27.3
Red clover (*Trifolium pratense* L.)	176	170	9	87	127	1	24	4	8	13	0	0	24.5	81.3	3.9
White clover (*Trifolium repens* L.)	55	74	51	55	7	72	11	11	60	3	0	8	13.1	26.0	27.2
Other legumes	43	0	0	0	0	3	0	0	0	0	0	0	10.5	-	1.3
Other botanical families	119	100	309	359	345	409	50	24	258	168	76	329	62.0	70.6	72.8
Northern dock (*Rumex longifolius* DC.)	49	31	66	98	50	68	15	8	140	38	55	86	28.6	14.0	44.1
Dandelion (*Taraxacum* spp.)	37	42	53	89	119	126	16	1	64	28	8	32	16.7	39.1	39.7
Common sorrel (*Rumex acetosa* L.)	4	0	14	79	66	28	1	0	0	57	0	65	32.4	36.8	29.5
Creeping buttercup (*Ranunculus repens* L.)	7	9	5	52	9	26	2	0	1	22	10	21	15.4	8.5	11.8
Meadow buttercup (*Ranunculus acris* L.)	5	8	42	11	79	30	2	4	38	4	0	86	3.0	41.3	33.1
Other spp.	5	10	129	32	22	130	17	10	25	20	3	74	12.2	11.1	40.3
Number of spp. per field	15	17	24	17	17	21	12	13	16	13	14	19	0.9	2.8	2.7

### Effects of Grassland Management, Production System and Season on Feed Intake, Milk Yield and Milk Gross Composition

Concentrate intake per cow and year and per 100 kg of ECM produced were on average 32% (*P* <0.001) and 24% (*P* = 0.001), respectively, lower on ORG-farms than on CON-farms (Table 2). Forage intake per cow and year was on average 10% higher (*P* = 0.02) on SG-farms than on LG-farms, whereas forage intake per 100 kg of ECM produced was 22% higher (*P* = 0.004) on ORG-farms than on CON-farms.

**Table 2:** Feed intake, milk production, milk gross composition and fertility indicators on dairy farms with organic production system (ORG) and short-term (ORG-SG) or long-term grassland management (ORG-LG) and dairy farms with conventional production system (CON) and short-term (CON-SG) or long-term grassland management (CON-LG).

Item	Farming system					*P*-value				
	ORG-SG	ORG-LG	CON-SG	CON-LG	SEM	G^1^	P(G)^2^	ORG-SG vs. ORG-LG^3^	CON-SG vs. CON-LG^4^	ORG vs. CON^5^
n	14	14	14	14						
Feed intake										
Forage, GJ NEL^6^/year and cow	23.9	21.9	22.8	20.0	0.99	0.02	0.31	0.20	0.04	0.15
Forage, GJ NEL/100 kg ECM^7^	0.362^a^ ^b^	0.376^a^	0.295^b^	0.312^a^ ^b^	0.0204	0.47	0.02	0.67	0.54	0.004
Concentrates, GJ NEL/year and cow	9.70^b^	8.55^b^	13.43^a^	13.53^a^	0.903	0.57	<0.001	0.38	0.94	<0.001
Concentrates, GJ NEL/100 kg ECM	0.141^b^	0.149^b^	0.173^a^ ^b^	0.208^a^	0.0122	0.09	0.003	0.65	0.05	0.001
Milk production and milk gross composition										
Lactating cows, adjusted to 305 d lactation	21.7	15.0	18.3	17.5						
Milk quota, tonnes	137	87	119	122						
Milk delivery, tonnes	129	72	125	107						
ECM yield, kg/year and cow	6,814^a^ ^b^	5,855^b^	7,787^a^	6,573^a^ ^b^	331.2	0.003	0.05	0.05	0.02	0.02
Milk yield, kg/year and cow	6,621^a^ ^b^	5,647^b^	7,550^a^	6,448^a^ ^b^	305.7	0.002	0.03	0.03	0.02	0.009
Fat, g/kg milk	4.11	4.35	4.17	4.11	0.202	0.66	0.68	0.40	0.82	0.66
Protein, g/kg milk	3.49^a^	3.29^c^	3.43^a^ ^b^	3.33^b^ ^c^	0.033	<0.001	0.33	<0.001	0.04	0.80
Lactose, g/kg milk	4.71	4.64	4.69	4.70	0.032	0.22	0.12	0.03	0.60	0.42
Fertility indicators										
Age at first calving, month	25.8	26.6	25.3	25.1	0.65	0.64	0.23	0.38	0.82	0.12
Calving interval, month	12.9	13.4	12.4	12.5	0.39	0.30	0.09	0.31	0.76	0.03
Cows culled due to fertility problems, number/total number of cows	0.073	0.068	0.103	0.160	0.0278	0.36	0.07	0.91	0.16	0.04

^1^ G = Effect of grassland management.

^2^ P(G) = Effect of production system within grassland management.

^3^ Contrast of ORG-SG vs. ORG-LG.

^4^ Contrast of CON-SG vs. CON-LG.

^5^ Contrast of ORG vs. CON.

^6^ Net energy lactation.

^7^ Energy corrected milk

^a, b, c^ Means within a row with different letters differ (Tukey-Kramer test, *P* < 0.05).

Milk yield per cow and year was 12% lower (*P* = 0.009) on ORG-farms than on CON-farms. The cows on SG-farms yielded on average 15% more (*P* = 0.002) milk with 4% more (*P* <0.001) protein than the cows on LG-farms. Lower milk concentrations of fat and higher concentrations of lactose were found in the indoor-feeding periods than the outdoor-feeding periods [17].

### Effects of Grassland Management, Production System and Season on Phytoestrogen Concentrations in Milk

Compared with milk from ORG-LG, milk from ORG-SG had higher concentrations of biochanin A (*P* = 0.05), genistein (*P* = 0.003), formononetin (*P* <0.001), daidzein (*P* <0.001), equol (*P* <0.001) and sum of isoflavones (*P* <0.001) (Table 3). No differences were found between CON-SG and CON-LG. ORG-farms had higher milk concentrations of genistein (*P* = 0.003), formononetin (*P* <0.001), daidzein (*P* <0.001), equol (*P* <0.001) and sum of isoflavones (*P* <0.001). Milk sampled during the indoor-feeding periods had higher concentrations of biochanin A (*P* = 0.02), daidzein (*P* = 0.03), equol (*P* <0.001) and sum of isoflavones (*P* <0.001). Equol was the isoflavone found in highest concentrations in milk samples from all farming systems with concentrations varying between 0 and 644 μg/kg milk. Grassland management, production system or season did not affect Prunetin.

**Table 3:** Phytoestrogen concentrations in milk on dairy farms with organic production system (ORG) and short-term (ORG-SG) or long-term grassland management (ORG-LG) and dairy farms with conventional production system (CON) and short-term (CON-SG) or long-term grassland management (CON-LG) during indoor (IN) and outdoor-feeding periods (OUT).

Phytoestrogen, μg/kg milk	Farming system					Season			*P*-value								
	ORG-SG	ORG-LG	CON-SG	CON-LG	SEM	IN	OUT	SEM	G^1^	P(G)^2^	M^3^	G×M^4^	P(G) ×M^5^	ORG-SG vs. ORG-LG^6^	CON-SG vs. CON-LG^7^	ORG vs. CON^8^	IN vs. OUT^9^
n	42	42	42	42		112	56										
Isoflavones																	
Biochanin A	1.3	0.5	0.4	0.5	0.26	0.8	0.5	0.21	0.23	0.07	<0.001	0.05	0.94	0.05	0.75	0.09	0.02
Genistein	4.2^a^	3.4^b^	3.4^b^	3.1^b^	0.17	3.5	3.5	0.16	0.005	0.004	0.16	0.08	0.76	0.003	0.28	0.003	0.98
Formononetin	6.8^a^	3.9^b^	3.7^b^	3.3^b^	0.34	4.6	4.1	0.34	<0.001	<0.001	0.004	0.31	0.14	<0.001	0.49	<0.001	0.06
Daidzein	5.2^a^	2.6^b^	2.1^b^	1.6^b^	0.35	3.1	2.5	0.34	0.001	<0.001	0.26	0.09	0.37	<0.001	0.29	<0.001	0.03
Prunetin	1.3	1.1	0.8	0.9	0.23	1.0	1.1	0.20	0.76	0.31	0.65	0.20	0.93	0.58	0.89	0.15	0.34
Equol	240.5^a^	65.4^b^	53.3^b^	34.1^b^	20.33	122.6	49.8	19.22	<0.001	<0.001	<0.001	0.12	0.19	<0.001	0.51	<0.001	<0.001
Sum isoflavones	259.2^a^	76.8^b^	63.7^b^	43.5^b^	21.08	135.5	61.4	19.80	<0.001	<0.001	<0.001	0.11	0.18	<0.001	0.51	<0.001	<0.001
Lignans																	
Secoisolariciresinol	12.2^a^	12.2^a^ ^b^	10.7^b^	11.6^a^ ^b^	0.41	12.5	10.0	0.49	0.37	0.01	<0.001	0.12	0.29	0.96	0.14	0.008	<0.001
Matairesinol	0.7	0.7	0.7	0.7	0.07	0.9	0.3	0.08	0.72	0.96	<0.001	0.60	0.98	0.65	0.96	0.99	<0.001
Enterodiol	0.5^a^	0.3^a^ ^b^	0.3^b^	0.3^a^ ^b^	0.05	0.3	0.4	0.05	0.37	0.007	<0.001	0.10	0.87	0.09	0.77	0.006	0.01
Enterolactone	100.7^a^	79.7^a^ ^b^	62.8^b^	66.4^a^ ^b^	10.25	53.1	126.0	10.59	0.51	0.001	<0.001	0.61	0.07	0.16	0.80	<0.001	<0.001
Sum lignans	114.0^a^	90.6^a^ ^b^	74.4^b^	78.9^a^ ^b^	10.69	66.8	134.9	10.84	0.49	<0.001	<0.001	0.67	0.09	0.14	0.77	<0.001	<0.001
Coumestans																	
Coumestrol^10^	0.8	0.5	0.1	0.3	0.40	0.3	0.7	0.28	0.87	0.45	0.30	0.66	0.35	0.53	0.69	0.31	0.13

^1^ G = Effect of grassland management.

^2^ P(G) = Effect of production system within grassland management.

^3^ M = Effect of month.

^4^ G×M = Interaction between grassland management and month.

^5^ P(G)×M = Interaction between production system within grassland management and month.

^6^ Contrast of ORG-SG vs. ORG-LG.

^7^ Contrast of CON-SG vs. CON-LG.

^8^ Contrast of ORG vs. CON.

^9^ Contrast of IN vs. OUT.

^10^ Not analyzed in 2008; only month 2, 6 and 10 in 2007 included; n (farming system) = 21, n (IN) = 56, n (OUT) = 28.

^a, b^ Means within a row with different letters differ (Tukey-Kramer test, *P* < 0.05).

**Fig 1:**
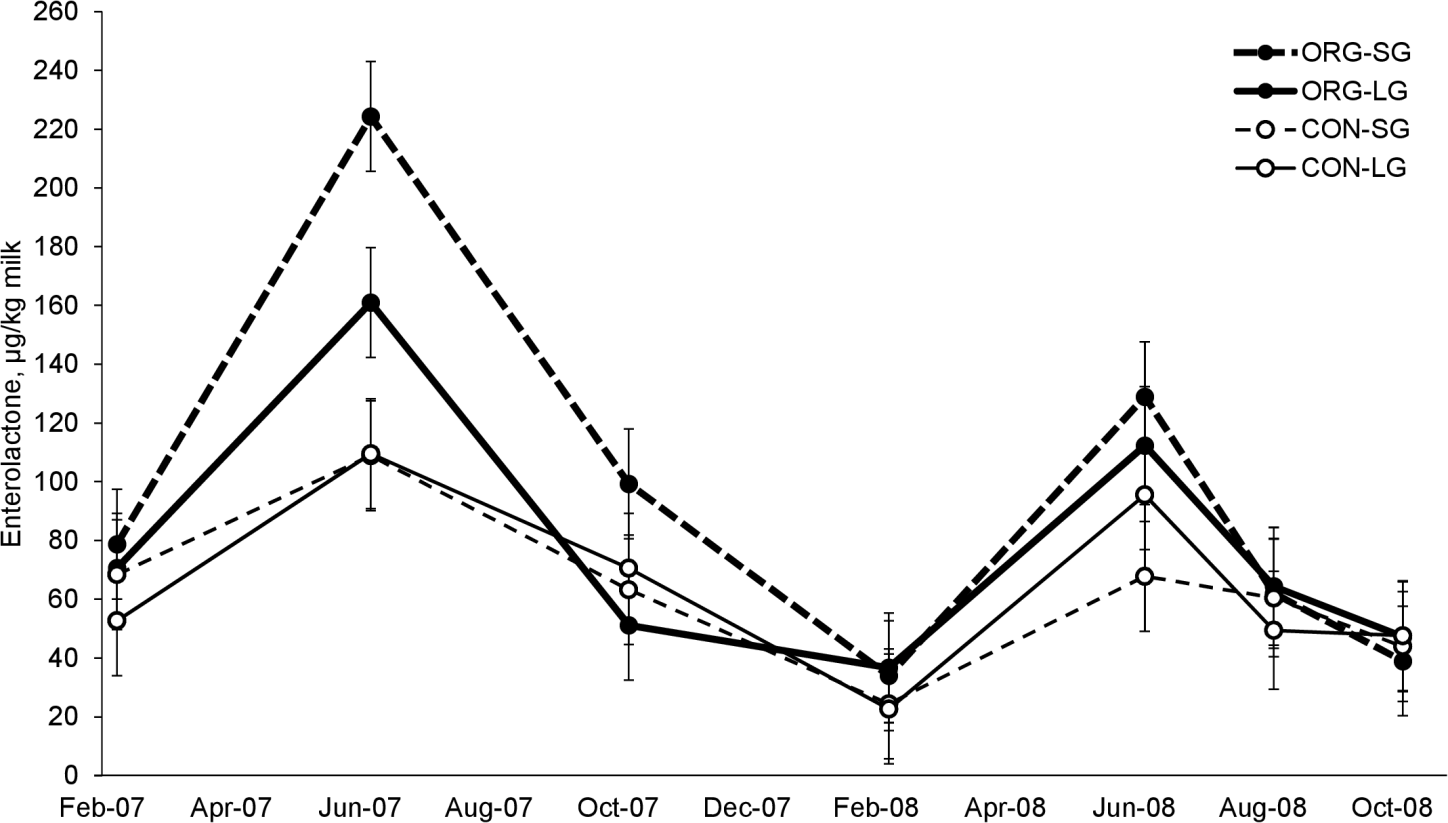
Seasonal variation of enterolactone concentrations in milk. Mean values in bulk-tank milk from dairy farms with organic production system and short-term (ORG-SG) or long-term grassland management (ORG-LG) and dairy farms with conventional production system and short-term (CON-SG) or long-term grassland management (CON-LG) in indoor (n = 7, error bars indicate standard error of the mean).

Grassland management did not affect milk concentrations of total or individual lignans. Compared with milk from CON-farms, milk from ORG-farms had higher concentrations of secoisolariciresinol (*P* = 0.008), enterodiol (*P* = 0.006), enterolactone (*P* <0.001) and total lignans (*P* <0.001). Milk concentrations of secoisolariciresinol (*P* <0.001) and matairesinol (*P* <0.001) were higher during the indoor-feeding periods, whereas the concentrations of enterodiol (*P* = 0.01), enterolactone (*P* <0.001) and total lignans (*P* <0.001) were higher during the outdoor-feeding periods. However, in August 2008 the concentrations of enterolactone were lower than in June and similar to the indoor feeding periods (Fig 1). Enterolactone was the lignan found in highest concentrations in milk samples from all farming systems (9 to 335 mg/kg milk).

Coumestrol was not affected by grassland management, production system or season.

### Correlations between Herbage Botanical Composition and Phytoestrogens in Milk

**Fig 2:**
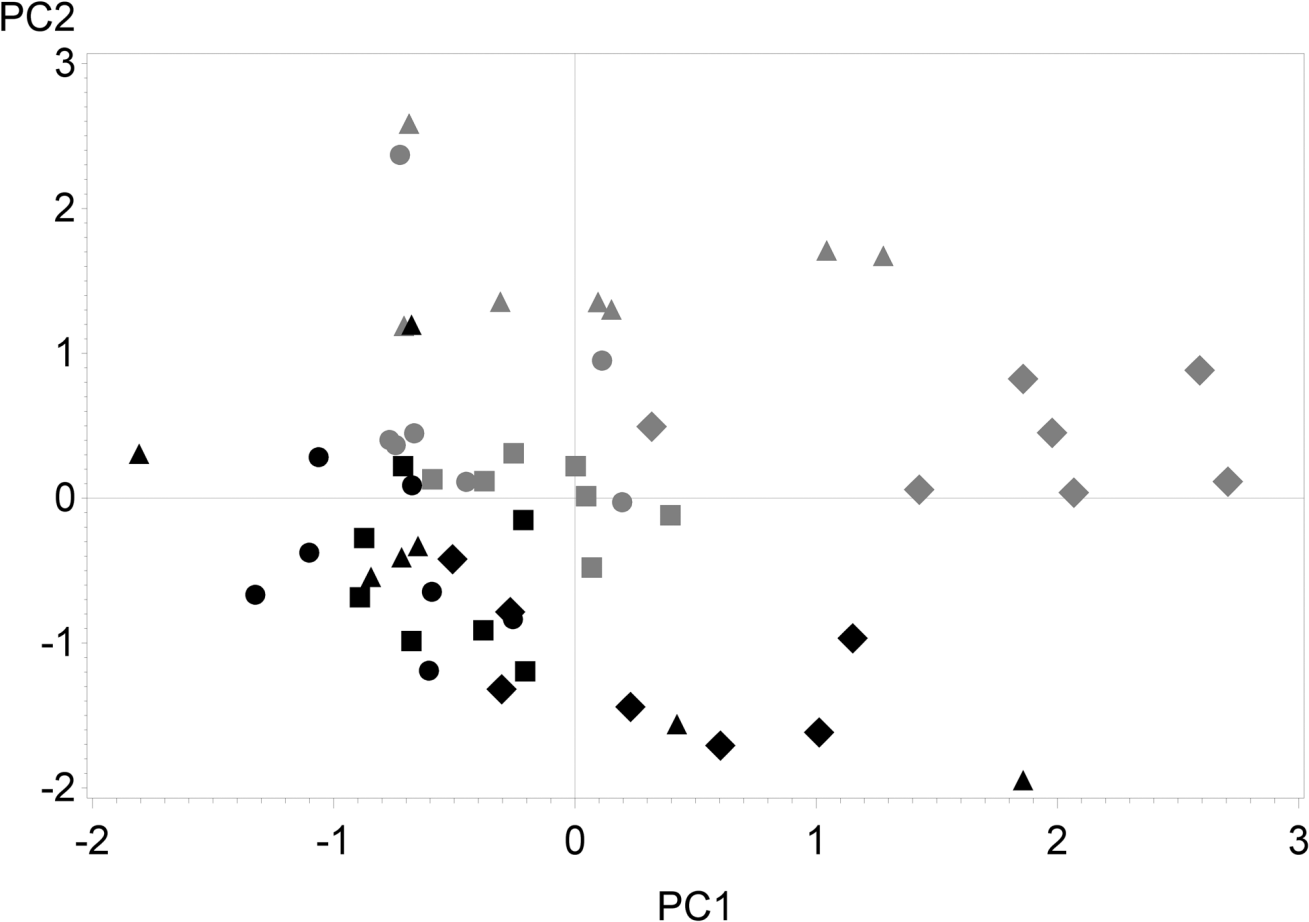
Score plot of 28 dairy farms in indoor and outdoor-feeding periods. Score plot for first and second principal component (PC1 vs. PC2) for dairy farms with organic production system and short-term (ORG-SG, ◯) or long-term grassland management (ORG-LG, △) and dairy farms with conventional production system and short-term (CON-SG, ☐) or long-term grassland management (CON-LG, ◇) in indoor (grey: February and October) and outdoor-feeding (black: June) periods, based on variables of milk phytoestrogen concentrations, herbage proportions of botanical families and cows’ daily concentrate DMI (means over 2 yr for 28 farms).

**Fig 3:**
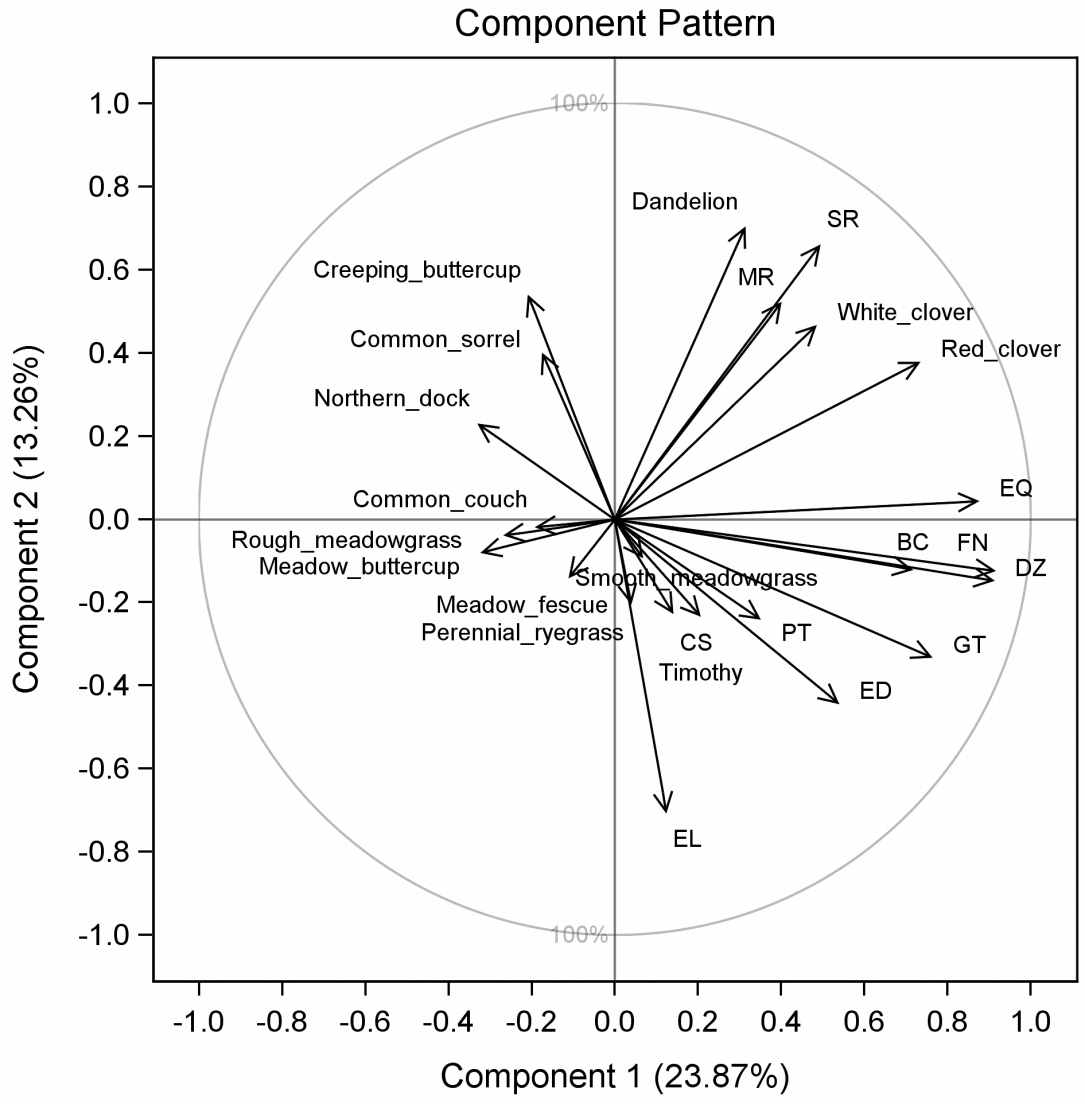
Correlation loading plot phytoestrogen concentrations in bulk-tank milk, herbage botanical composition and concentrate intake. Correlation loading plot for first and second principal component (PC1 vs. PC2) showing the relationship between milk concentrations of the phytoestrogens biochanin A (BCA), genistein (GT), formononetin (FN), daidzein (DZ), prunetin (PT), equol (EQ), secoisolariciresinol (SR), matairesinol (MR), enterodiol (ED), enterolactone (EL) and coumestrol (CS); herbage proportions of the predominant plant species; and cows’ concentrate DMI (means of February and October (indoor-feeding periods) and means of June (outdoor-feeding periods) over 2 yr for 28 farms).

In the principal component analysis of herbage botanical composition, concentrate DMI and milk concentrations of phytoestrogens, the principal component 1 explained 23%, and the principal component 2 explained 13% of the total variation (Figs 2 and 3). Together, the principal components 1 and 2 separated most of the indoor-feeding samples from the outdoor-feeding samples. The distances between clusters of indoor- and outdoor-feeding samples were larger for ORG-farms than for CON-farms.

The herbage proportion of red clover was positively correlated with the proportions of white clover (r = 0.51; *P* <0.001) and dandelion (r = 0.47; *P* <0.001) and the milk concentrations of biochanin A (r = 0.48; *P* <0.001), genistein (r = 0.36; *P* = 0.007), formononetin (r = 0.58; *P* <0.001), daidzein (r = 0.58; *P* <0.001), equol (r = 0.82; *P* <0.001), secoisolariciresinol (r = 0.47; *P* <0.001) and matairesinol (r = 0.40; *P* = 0.002). Furthermore, smooth meadowgrass was negatively correlated with secoisolariciresinol (r = 0.26; *P* = 0.049) and matairesinol (r = 0.24; *P* = 0.074), and rough meadowgrass (*Poa trivialis* L.) was negatively correlated with formononetin (r = 0.34; *P* = 0.009) and equol (r = 0.27; *P* = 0.047). Northern dock (r = 0.28; *P* = 0.035) and meadow buttercup (r = 0.27; *P* = 0.045) were negatively correlated with genistein. No correlations were found between plant species and milk concentrations of enterodiol, enterolactone and coumestrol.

### Indicators of Fertility

Compared to CON-farms, ORG-farms had slightly longer (*P* = 0.03) calving intervals, but fewer (*P* = 0.04) cows were culled due to fertility problems. Age at first calving was similar for all farming systems. None of the fertility indicators were affected by grassland management.

## Discussion

### Botanical Composition of Diets

Differences in seed mixtures, harvesting managements, N-fertilization level and use of herbicides may explain the differences in botanical composition between farming systems. Legumes are important forage species in organically farmed grassland systems due to their N-fixation capability. In Norway, red clover is commonly included in organically grasslands intended for harvesting by cutting or a combination of cutting and grazing, whereas white clover is often included in pastures. In conventionally managed grasslands N-fertilizers boost plant growth in early spring favoring the growth of grasses in preference to legumes. Other plant species may have been favored by absence of herbicides on organically managed grasslands, long grassland duration on LG-farms or grazing animals in all systems.

### Effects of Grassland Management, Production System and Season on Phytoestrogen Concentrations in Milk

#### Isoflavones

High concentrations of equol in milk from ORG-SG farms is in accordance with other studies where cows were fed red clover containing silage [19–21] or grazing red clover containing pastures [22, 23]. In the studies by Andersen et al. [23] and Steinshamn et al. [19], the concentrations of equol were similar to the present study, whereas in the studies by Höjer et al. [21] (experiment 1: 1,494 μg/kg, experiment 2: 716 μg/kg) and Adler et al. [22] (1,199 μg/kg), the concentrations were considerably higher than in the present study. High concentrations of equol in milk are related to high intake of the precursors formononetin and daidzein [18]. Formononetin is found in high concentrations in red clover [30] and thus the proportion of red clover in the forage is an important factor. Furthermore, the concentrations of formononetin in red clover depend on plant phenological stage, leaf-stem ratio and season [31–32]. In the studies of Andersen et al. [20, 23], Höjer et al. [21], Adler et al. [22] and Steinshamn et al. [19], the formononetin concentrations in red clover grass silage or pasture ranged from 2,790 to 11,420 mg/kg DM and concentrations in white clover grass silage or pasture ranged from 142 to 201 mg/kg DM. Unfortunately, it was not possible to measure red clover intake and concentration of phytoestrogens in the herbage in the present study.

On the farming system level, similar differences as in the present study have been found between organic and conventional farms. Hoikkala et al. [25] found 411 μg/L of equol in organic skimmed milk whereas conventionally produced milk contained 62 μg/L of equol. Milk samples were collected between January and March and represent milk produced during the indoor-feeding periods. Also Antignac [24] found higher concentrations of equol in organic milk (191.0 μg/L) than conventional milk (36.4 μg/L), but the sampling time of the milk samples is not available.

In the present study, less red clover was found in pastures compared to fields that were cut or cut and grazed in combination, and differences were greater on ORG-farms than on CON-farms. Thus, it is likely that the red clover proportion in the herbage DMI was higher during the indoor-feeding periods than the outdoor-feeding periods. Herbage botanical composition was estimated before first cut in 2007. In organic farming, red clover often makes up a higher proportion in the herbage yield of the second cut than of the first cut [33]. Therefore, red clover intake was most likely higher on ORG-farms than what could have been expected based on the botanical composition before the first cut. It has been shown that including concentrate in the diet decreases equol concentrations in milk slightly [19]. Thus, the lower concentrate intake in cows on ORG-farms compared to CON-farms may partly explain a part of the differences in milk equol concentrations. The observed differences in milk yield may also have affected the recovery of isoflavones in milk.

Phytoestrogen concentrations in feed were not analyzed in the present study, but based on reported concentrations in other studies the contribution of forages and concentrates can be estimated. Soybeans (*Glycene max* (L.) Merr.) have high concentrations of daidzein (105 to 560 mg/kg DM), but only small amounts of formononetin [7]. Typically, concentrations of formononetin in silages including red clover are 2,500 to 3,000 mg/kg DM, in silages with white clover about 150 mg/kg DM and in grass based silages <50 mg/kg DM, whereas concentrations of daidzein are much lower [19–21, 34]. Inclusion of soybeans in concentrate mixtures was on average 4% and 12% in organic and conventional production, respectively. In addition, the concentrate level was higher on organically than on conventionally managed farms. Thus, the intake of the equol precursors from concentrate was likely much higher on CON-farms than ORG-farms. Thus, in organically produced milk, equol derives primarily from the forage, whereas in conventionally produced milk a large share derives from soybeans.

#### Lignans

The concentrations of lignans in milk were not associated with grassland management, but with production system and season. Unlike the situation for isoflavones, the reasons for the observed effects on lignans are not clear. Compared with milk from CON-farms, milk from ORG-farms had more secoisolariciresinol, enterodiol and enterolactone. A possible reason is that the diets fed on ORG-farms contained higher levels of mammalian lignan precursors such as secoisolariciresinol, mataresinol, lariciresinol and pinoresinol [35]. High concentrations of these precursors are found in flaxseed and sesame seed, but also brassica vegetables contain significant concentrations. However, brassicas were only found in small proportions (Shepherd's-purse, *Capsella bursa-pastoris* (L.) Medik.). Milk from the indoor-feeding periods contained more secoisolariciresinol and mataresinol, but less enterodiol and enterolactone than milk from the outdoor-feeding periods. The higher concentrations of enterodiol and enterolactone in milk from the outdoor-feeding periods indicate that plant species rich in mammalian lignan precursors may be found in higher proportions in pastures than fields cut or cut and grazed in combination. These may be unsown grasses or non-legume dicotyledons such as northern dock and dandelion. However, the principal component analysis of herbage botanical composition and milk concentrations of phytoestrogens did not show any correlations between enterolactone concentrations in milk and plant species. The low concentrations in August 2008 indicate that the intake of enterolactone precursors may depend on the phonological stage of forage plants. The high concentrations in June may be related to the lignification process during the elongation stage in grasses. Furthermore, the stability of mammalian lignan precursors in silage may have affected the intake of lignans.

Steinshamn [19] found positive effect of concentrate on enterolactone concentrations in milk. This effect may have decreased the differences between ORG-farms and CON-farms caused by differences in forage intake. In contrast, Antignac et al. [24] found no effect of productions system on enterolactone.

#### Botanical Composition and Phytoestrogens in Milk

The proportion of red clover in the herbage is positively correlated with milk concentrations of the mammalian isoflavone equol. Therefore, organically produced bulk-tank milk contained more equol than conventionally produced milk, and milk from ORG-SG farms had more equol than milk from ORG-LG farms. Milk produced in the indoor-feeding periods had more equol than milk produced in the outdoor feeding period, because pastures contained less red clover than fields intended for silage production. Organically produced milk had also higher concentrations of the mammalian lignin enterolactone, but in contrast to equol, concentrations increased in the outdoor-feeding periods compared to the indoor-feeding periods.

#### Phytoestrogens and Fertility in Dairy Cows

ORG-SG farms who had the highest proportions of red clover in the forage and the highest milk concentrations of phytoestrogens did not differ from ORG-LG farms in terms of the fertility indicators. The red clover proportion before first cut in 2007 was 17.4% for ORG-SG and even if a higher proportion can be assumed for the second cut, the average intake of red clover may be only moderate explaining no observation of impaired fertility on these farms relative to the others. The slightly longer calving intervals on ORG-farms, may be due to lower plane of nutrition as indicated in a previous field study on organic dairy farms [36]. Higher culling frequency on conventional farms may be due to higher production aims. In conclusion, neither intake of red clover or phytoestrogen concentrations in milk can be related to fertility issues in the present study.

This study shows that production system, grassland management and season affect milk concentrations of phytoestrogens. However, compared to soy products, milk concentrations of phytoestrogens are low and future studies are required to investigate if the intake of phytoestrogens from dairy products have physiologic effects in humans.
